# Correction: Enhancing scanning electrochemical microscopy's potential to probe dynamic co-culture systems *via* hyperspectral assisted-imaging

**DOI:** 10.1039/d2an90050e

**Published:** 2022-06-29

**Authors:** Sondrica Goines, Mingchu Deng, Matthew W. Glasscott, Justin W. C. Leung, Jeffrey E. Dick

**Affiliations:** Department of Chemistry, The University of North Carolina at Chapel Hill Chapel Hill NC 27599 USA jedick@email.unc.edu; Department of Radiation Oncology, College of Medicine, University of Arkansas for Medical Sciences Little Rock AR 72205 USA; Lineberger Comprehensive Cancer Center, The University of North Carolina at Chapel Hill Chapel Hill NC 27599 USA

## Abstract

Correction for ‘Enhancing scanning electrochemical microscopy's potential to probe dynamic co-culture systems *via* hyperspectral assisted-imaging’ by Sondrica Goines *et al.*, *Analyst*, 2022, **147**, 2396–2404, https://doi.org/10.1039/D2AN00319H.

The authors regret that the citations to references 15, 27 and 28 in the sub-section “Variable fluorescence bandpass hyperspectral imaging of Hep G2 cells with correlated scanning electrochemical microscopy” are incorrect. The references should be given as 17, 37 and 42 and should be cited on page 2399.

Reference 42 was not included in the original article and is listed below.

The Royal Society of Chemistry apologises for these errors and any consequent inconvenience to authors and readers.

**References**

42. A. Schulte and W. Schuhmann, *Electrochemical Methods for Neuroscience*, 2007, **1**, 353–372.

## Supplementary Material

